# Isotopic composition of individual hydrobiidae gastropods from neotropical lakes Esmeralda and Chichancanab in the Maya Cochuah region, Mexico: implications for palaeolimnological research

**DOI:** 10.1007/s10933-026-00385-3

**Published:** 2026-03-19

**Authors:** Haydar B. Martinez-Dyrzo, Matthew D. Jones, Sarah E. Metcalfe, Melanie J. Leng, Roger Medina-Gonzalez

**Affiliations:** 1 School of Geography, University of Nottingham, Nottingham, NG7 2RD UK; 2 Instituto de Geología, Universidad Nacional Autónoma de México, Av. Universidad 3000, Ciudad Universitaria, Coyoacan, 04510 Mexico City, Mexico; 3 NERC Isotope Geosciences Laboratory, British Geological Survey, Keyworth, Nottingham, NG12 5GG UK; 4 Licenciatura en Biología. Facultad de Medicina Veterinaria y Zootecnia. Campus de Ciencias Biológicas y Agropecuarias, Universidad Autónoma de Yucatán, Km 15.5 Carretera Mérida, Xmatkuil, Yucatán México

**Keywords:** Palaeoclimate reconstruction, Gastropod shells, Stable Isotopes, Maya lowlands, Yucatan Peninsula

## Abstract

Most lake-carbonate-isotope records from the Maya Lowlands are based on picking fossil carbonate ostracods and gastropods, often using the species *Pyrgophorus coronatus,* and then combining individual specimens from a single core layer to provide a composite sample. What has been left relatively unexplored is the variability between individual shells and the impact this might have on the values obtained from such composite samples. Here, the stable carbon and oxygen isotope signatures of modern *P. coronatus,* a littoral and detritivore species, and other Hydrobiidae taxa (*Aroapyrgus* sp. and *Tryonia* sp.) found in Lake Esmeralda and its much larger sister lake Chichancanab, in the Northern Maya Lowlands (also called Mayab or Yucatan Peninsula), were analysed to explore some of the environmental variables that might affect isotope composition. In addition, the carbon and oxygen isotope values derived from individual downcore gastropod shells were compared with those from bulk fine-fraction carbonate sediment from a core from Lake Esmeralda covering the last c. 6600 years. The results show that the median oxygen isotope value of a set of shells collected for a single stratigraphic layer tends to be about the same as the bulk sediment, even though their individual values can present a bimodal distribution, which might represent changes between dry and rainy seasons across the sample’s period. In addition, no significant difference in the oxygen isotope values was found in using a particular Hydrobiidae taxon over another, indicating a lack of significant vital effects. The carbon isotopic signature of modern shells varies with location within the lake, and shows relatively little variability down core, suggesting non-climatic factors may be more dominant in controlling shell carbonate δ^13^C values in these systems.

**Supplementary Information:**

The online version contains supplementary material available at 10.1007/s10933-026-00385-3.

## Introduction

Oxygen and carbon isotope (δ^18^O and δ^13^C) records have a pivotal role in understanding climate change across the Maya Lowlands (Mayab), being applied to lake sediments and speleothems, and largely focusing on water balance and precipitation amount reconstructions respectively (Douglas et al. 2016a, b; James et al. 2025). Lake-carbonate-isotope records from the Maya lowlands have mainly been produced from gastropod shells, combining individuals (n ≥ 15) from a single layer to provide a composite sample (Curtis et al. 1996; Hodell et al. 1995, 2005a, b; Wahl et al. 2014), avoiding the potential impact that in-washed material from the carbonate-dominated catchments might have on bulk-sediment analysis. What has been left relatively unexplored, other than Escobar et al. (2010), is the variability between individual shells and the potential impact this might have on the values obtained from a composite sample.

Methodologies involving gastropod shells present some challenges in getting a clear isotope signal, including the need to (1) select a particular taxon of gastropods which will be ubiquitously present in the sedimentary record due to potential different vital effects between species (Apolinarska et al. 2015b; Holmes and Chivas 2002; Leng and Marshall 2004); (2) establish the number of individual shells needed to obtain a reliable isotopic value for a particular stratigraphic horizon (Apolinarska et al. 2015a; Escobar et al. 2010), and (3) assess the dependence of the individual gastropod’s isotope composition on habitat (Apolinarska et al. 2015c) given potential variable lake water δ^18^O and temperature in space and time (Jones et al. 2002).

*Pyrgophorus* (assumed to be *P. coronatus*) has been widely selected for isotope studies in lakes in the Maya area (Carrillo-Bastos et al. 2013; Covich 1976; Covich and Stuiver 1974; Curtis et al. 1996, 1998; Hodell et al. 2005a, b; Metcalfe et al. 2009; Wahl et al. 2014). Some studies, however, have focused on the whole Hydrobiidae family, to which the genus *Pyrgophorus* belongs, without distinguishing between genus or species (Whitmore et al. 1996). *Pyrgophorus coronatus* presents two morphologies, a smooth and spinose form (Dillion 2006). The spinose form is the only one that can sensu stricto be associated with *P. coronatus*. The taxonomic status of most of the nominal species called "*Pyrgophorus*" is uncertain (Hershler 1992). For instance, *Pyrgophorus platyrachis* could be an ecophenotype of *P. coronatus* (Andrew et al. 2002). Both forms have, however, been commonly associated with this species in the Maya area (Carrillo-Bastos et al. 2013; Covich 1976; Covich and Stuiver 1974; Curtis et al. 1998, 1996; Hodell et al. 2005a, b; Metcalfe et al. 2009; Wahl et al. 2014), so both forms are identified as *P. coronatus.*

Escobar et al. (2010) investigated the optimum number of individual shells from a given core sample required to obtain reliable reconstructions of low-frequency (decadal/centennial) climate variability in Lake Chichancanab, where the first regional high-resolution isotope-carbonate-shell record was developed (Hodell et al. 1995). They calculated that for δ^18^O, between 1 and 45 shells of *P. coronatus* were needed per sample level. Similar work from outside the region, in Germany, has also pointed to a similar order of magnitude of individual shells (≥ 15) as an optimum to represent the range in δ^18^O and δ^13^C for a given time period (Apolinarska et al. 2015a).

Hydrobiidae gastropods can live from a few months to more than a year. Therefore, the isotope record in their shells may reflect seasonal or annual means. *Pyrgophorus coronatus* is a littoral form and a detritivore (Dillion 2006), so in lakes with an extensive littoral area, it might show considerable spatial variability in its isotopic composition. Little is known about the ecology and life history of *P. coronatus* and other Hydrobiidae found in the Mayab. However, a study of Hydrobiidae in the San Francisco de Asís Lagoon System in South Spain, a coastal lagoon, indicates that the life span of Hydrobiidae species is up to 2 years, but many live 1.5 years (Drake and Arias 1995). To date, no similar study has been undertaken in the Mayab. In the Mexican context, over 1.5 years each snail would capture either two dry seasons or two wet seasons, potentially biasing the data in the shell (assuming uniform growth) to one season.

This research aims to further test the reliability of extracting a climatic signal using δ^18^O from gastropod shells in the Yucatan Peninsula. Subfossil shells, collected from the sediment–water interface, and modern water samples are used to see whether the δ^18^O and δ^13^C values are in equilibrium with contemporary environmental conditions, and whether isotope values vary between species, and spatially within a lake. These subfossil shell samples are then used to look again at variability in single shell δ^18^O and δ^13^C values, and how this may influence multi-shell samples. Finally, a down-core gastropod isotope record from Lake Esmeralda was compared against the isotope composition of the fine fraction carbonates from the same core. These approaches further our understanding of carbonate-isotope systems, particularly for gastropod shells, and their potential in proxy studies of past lake change.

### Study sites

**Fig. 1:**
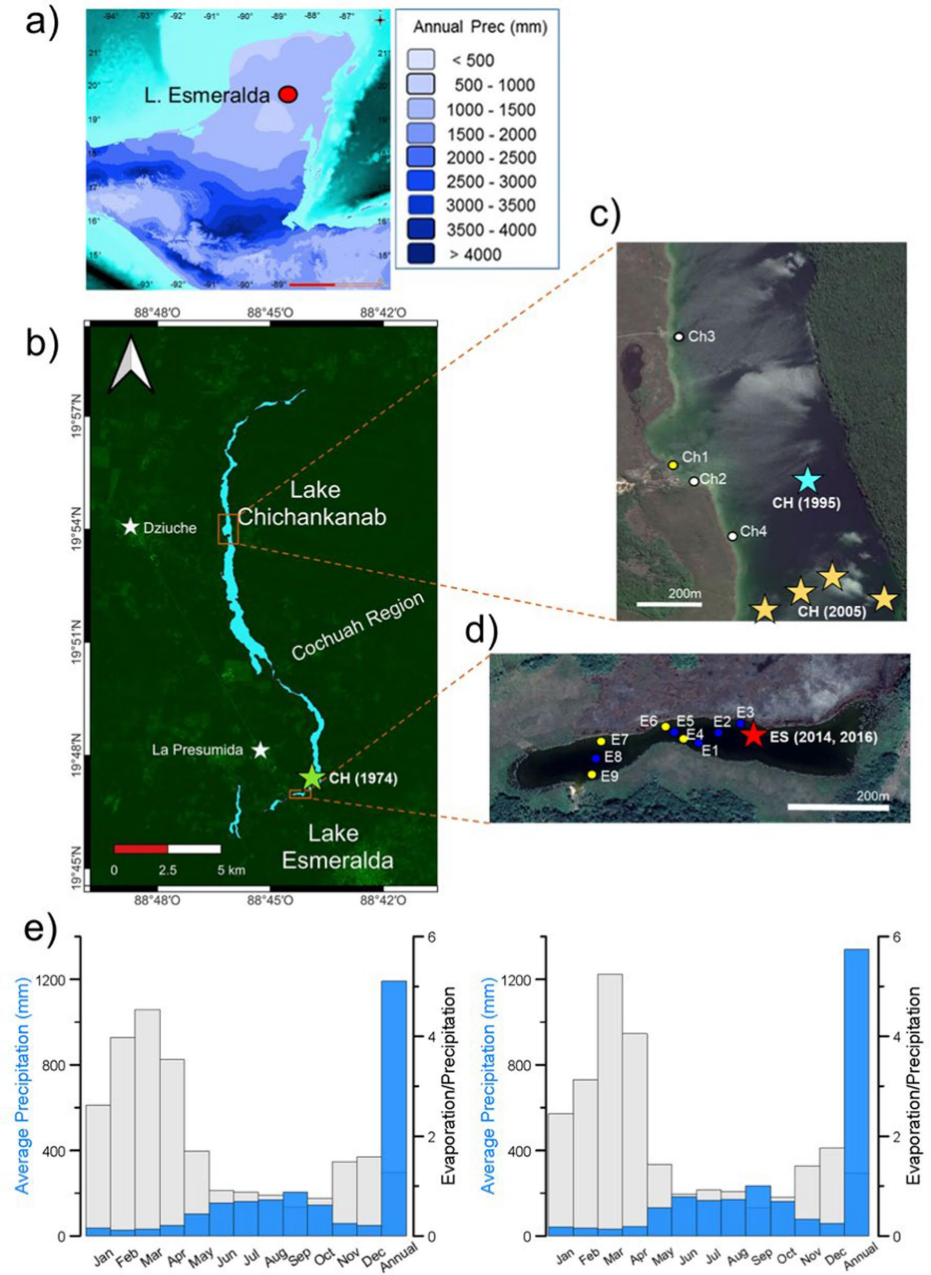
Context of Lake Esmeralda within the annual precipitation map (1950–2000) of the Maya region (a; after Nooren et al. 2018). Relative location of Lake Esmeralda to Lake Chichancanab (b; after INEGI 2021) and details of both lakes (c and d; after Google Earth Pro 7.3.6 2017). Meteorological stations (white stars), shell collection sites (white circles) and water and shell-collection sites (yellow circles) and water only collection sites (dark blue circles) are indicated for both lakes. The coring sites at Chichancanab used by Hodell et al. (1995) (pale blue star); Hodell et al. (2005a, b) (yellow stars) including CH1 7-III-04; and Covich and Stuiver (1974) (estimated location pale green star) as well as the site of cores ES-14 and ES-16–01, -02 (this study) (19° 46′ 59.9" N, 88° 44′ 10.7" W) (red star) in Lake Esmeralda. Average measured monthly and annual precipitation (blue) and calculated evaporation/precipitation (grey) from 1950 to 2010 at Dziuche CONAGUA (2010a) and La Presumida CONAGUA (2010b) (e)

Lake Esmeralda (c. 0.64 km long, 0.10 km wide, 4 m.a.s.l., maximum measured depth 3.5 m) lies immediately to the south of Lake Chichancanab (14.5 km long, 0.7 km wide, maximum depth 15 m) (Fig. 1) in the ancient Maya Cochuah region in the Mayab (Molina-Solís 1896).

Lake Esmeralda has been described as the 'sister lake' of Lake Chichancanab and it has been suggested that the two might join up during periods of high lake levels (Hodell et al. 2005a). Whilst Lake Chichancanab has been the focus of palaeoclimatic study from the initial work of Covich and Stuiver (1974) to the studies of Hodell et al. (1995, 2001, 2005a) and the more recent work of Douglas et al. (2014, 2016b) and Evans et al. (2018), there has been little published work on Lake Esmeralda (Covich and Stuiver 1974).

Average monthly precipitation (measured from 1950 to 2010) at the meteorological stations at Dziuche and La Presumida, near Chichancanab and Esmeralda, respectively (Fig. 1), allow modern regional climate to be described. The annual rainfall at Dziuche is 1191 mm (CONAGUA 2010a), similar to the precipitation measured at La Presumida, 1339 mm. The months with higher rainfall are between June to October (Fig. 1e), due to the NE Trade winds (moving northwards in response to the seasonal migration of the ITCZ), convective storms triggered by heating over the continent and tropical storms and hurricanes formed in the Tropical Atlantic which can affect the peninsula (Castro 2010; Gunn et al. 1995; Kappas 2009; Metcalfe et al. 2000; Montero-Serrano et al. 2011; Mosiño-Alemán and García-Acosta 1974). September is the wettest month (Fig. 1e). Precipitation at La Presumida (CONAGUA 2010b) shows some evidence for a period of reduced rainfall in July, known as the Canícula. This feature is unclear in the average conditions at Dziuche, but it occurs in some individual years (CONAGUA 2010a). At La Presumida mean annual temperature was 26.8 °C between 1991 and 2020, with the mean January temperature being 23.8 °C, and with a mean July temperature of 28.2 °C.

Lakes Esmeralda and Chichancanab are both CaSO_4_ dominated. Based on our own fieldwork and that of Perry et al. (2002) Esmeralda has a pH between 7.2 and 8.3, and conductivity of 2450 – 3995 µS cm^−1^, while Chichancanab has a pH between 7.93 and 8.45, with conductivity of 3462 – 4992 µS cm^−1^. Chichancanab is oligotrophic except near the sediment–water interface in deep water where it is eutrophic, while Esmeralda is mesotrophic except at the surface where it is eutrophic, based on Carlson and Simpson (1996). Water oxygen isotopes analysed by Perry et al. (2003) indicate that Lake Esmeralda is less evaporated than Chichancanab, with δ^18^O values between –2.40‰ in January and –1.80‰ in November, compared to the range of + 2.21 to + 4.40‰ in Lake Chichancanab (Evans et al. 2018; Metcalfe et al. 2022; Perry et al. 2003). This is consistent with the measured conductivity values.

## Material and methods

### Fieldwork

Samples of gastropod shells of the family Hydrobiidae were collected from the top 1–2 cm of unconsolidated surface sediments at four sites in Lake Esmeralda (E6 to E9) (Fig. 1d) and four sites in Lake Chichancanab (Ch1 to Ch4) using an Eckman Grab. The Chichancanab samples were all taken in water depths < 220 cm, so do not represent deeper parts of the lake (Fig. 1c).

Opportunistic spot samples for water isotopes were taken each January from Lake Chichancanab (since 2009) and Lake Esmeralda (since 2014). In January 2018, additional water samples for oxygen and hydrogen isotopes (δ^18^O/δD) and carbon isotopes (δ^13^C) of the dissolved inorganic carbon (DIC) were collected from the surface at nine sites in Lake Esmeralda designated as E1 to E9 (Fig. 1d). At E5 and E8 water samples were also collected at depth, 315 cm and 270 cm respectively. Surface water temperature was measured using a Hannah HI-98129 stick meter at the sites where samples were collected. In the northern hemisphere summer of 2024 additional surface water samples for δ^18^O and δD analysis were taken from Lake Esmeralda.

Three parallel cores ES-14 (4 drives), ES-16-01 (4 drives) and ES-16-02 (3 drives) were collected from the southern part of Lake Esmeralda at 19° 46′ 59.9" N, 88° 44′ 10.7" W in 2.63 m water depth (Fig. 1d) over two field seasons in January 2014 and 2016 using a Livingstone type corer with a 1 m core barrel. A Glew gravity core was also taken in 2016 to provide a larger sample of surface sediments. The cores were transferred to the University of Nottingham, UK, and stored at < 4 °C. Based on the distinctive visual stratigraphy of the Livingstone cores, a single combined 'master' sequence of 3.87 m was created.

### Gastropod-sample preparation and analysis

The sediment-surface, subfossil, gastropods were washed, to remove contaminants, using tap water, then distilled water and then in an ultrasonic bath with deionized water, and then classified into four taxa: *Aroapyrgus* sp., *Tryonia* sp., *Pyrgophorus coronatus* (smooth form), and *Pyrgophorus coronatus* (spinose form) according to the morphology of the shells (Dillion 2006). The classified shells were then rewashed in an ultrasonic bath for ten minutes with distilled water. Contaminants such as organic matter (including charcoal) and endogenic calcite were brushed off, and the shells were then treated again in the ultrasonic bath. The shells were dried in an oven for 24 h at 30 °C. Shells of similar size were selected for analysis, avoiding translucent shells that could belong to juveniles, as these were more likely to be at similar biological growth stages (Covich 1976, 2010).

Shell structure of a selection of individuals was investigated using a JEOL 6400 scanning electron microscope (SEM) in the low vacuum chamber filled with water vapour, at 80 Pa, a contrast = 40, a bias = 49.5 and a high voltage = 10 kV. Shells with evidence of diagenesis, dissolution and/or secondary carbonate growth, were classified according to the percentage of the total surface occupied by the diagenesis. The same samples were then observed using an Olympus SZX10 binocular optical microscope (20 × objective magnification). This confirmed that the diagenetic material observed using SEM could also be recognized using an optical microscope. This approach allowed all shells to be assessed relatively quickly, and individuals with diagenesis areas covering more than 10% of the shell surface to be rejected for further analysis. An initial assessment of the impact of the level of diagenesis on the isotope values measured showed there was no clear driver of isotope values by the amount of diagenesis observed (Martinez Izquierdo Dyrzo, 2021), however to minimise risk we took the decision to only include shells minimally impacted by diagenesis for use in the study.

XRD analysis of ten cleaned single shells from the sediment surface of Lake Esmeralda was conducted for determining their mineralogy using a Bruker D8 Advance diffractometer with a Bragg–Brentano geometry configuration and CuK_α_ source in the School of Chemistry, University of Nottingham.

The cleaned shells were sent to the British Geological Survey (BGS) for stable isotope analysis. They were plasma ashed to remove remnant organic material and crushed to a fine powder using an agate pestle and mortar. An aliquot of 30 mg was taken from each ground specimen (average shell weight is 50 mg), and 10 mg then used to determine δ^13^C and δ^18^O relative to VPDB in a GV Isoprime 100 mass-spectrometer with Multiprep (multi preparation system). An internal laboratory isotope standard KCM was measured to standardise and normalise the isotope data. The precision of the KCM was ≤  ± 0.05‰ for both δ^13^C and δ^18^O.

Ten stratigraphic layers, of 1 cm thickness, were selected from the Lake Esmeralda core sequence from which ten specimens, single shells of *P. coronatus*, were picked (Supplementary Table 1). Meanwhile, three stratigraphic layers were chosen for picking more than ten specimens of mixed Hydrobiidae. These layers, again of 1 cm thickness, were chosen randomly representing the top, middle and bottom of the sequence. The collected gastropods were cleaned and analysed using the same methodology as the subfossil shells.

### Water and sediment isotope analyses

The majority of the oxygen isotope (δ^18^O) measurements of water reported here were undertaken at the British Geological Survey and used the CO_2_ equilibration method with an Isoprime 100 mass spectrometer plus Aquaprep device. Deuterium isotope (δD) measurements used an online Cr reduction method with a EuroPyrOH-3110 system coupled to a Micromass Isoprime mass spectrometer. Isotope measurements used internal standards calibrated against the international standards VSMOW2 and VSLAP2. Uncertainties are typically ± 0.05‰ for δ^18^O, and ± 1.0‰ for δD.

The water samples taken in summer 2024 were analysed at the Facultad de Química, UNAM at the Parque Científico y Tecnológico de Yucatán. Samples were analysed using a Gasbench II sample preparation system coupled to a Thermo Scientific Delta-V Plus Isotope Ratio Mass Spectrometer. For δ^2^H analysis, 0.2 mL of sample was injected into a 12 mL vial containing a platinum catalyst, previously purged with a 2% H₂/He mixture. The samples were allowed to equilibrate for two hours at room temperature; then, an aliquot of H₂ was extracted and analysed. For δ^1^⁸O analysis, similarly, 0.2 mL of sample was injected into a 12 mL vial purged with a 0.3% CO₂/He mixture and then allowed to equilibrate for 20 h, also at room temperature. An aliquot of the isotopically equilibrated CO₂ was then extracted and its isotopic composition analysed. Sample analysis included certified VSMOW2 and SLAP2 standards, as well as internal standards LL1 and LL8. The maximum long-term analytical uncertainties of this technique are 0.2‰ for δ^18^O and 4‰ for δD.

Water δ^18^O values and water temperatures were used to calculate theoretical values for carbonate, both calcite and aragonite, using the equations proposed by Leng and Marshall (2004).

The fine carbonate fraction of the sediment from the stratigraphic layers was also sampled and sieved using a mesh size of 250 µm to remove most of the shell material. Organic material was removed (by oxidation with NaClO). Sample material was ground using an agate pestle and mortar and the equivalent of 10 mg of carbonate was reacted with anhydrous phosphoric acid in vacuum for 24 h at a constant 25 °C. The CO_2_ liberated was separated from water vapour under vacuum and collected for analysis. Measurements were made on a VG Optima mass spectrometer at the British Geological Survey. Overall analytical reproducibility for these samples is normally better than 0.2‰ for δ^13^C and δ ^18^O (2σ). The same method was also used for the ten samples collected from Lake Esmeralda for δ^13^C analysis on modern dissolved inorganic carbonate, precipitated from lake waters by adding BaCl_2_. The δ^13^C and δ^18^O values are reported as per mil (‰) deviations of the isotopic ratios (^13^C/^12^C, ^18^O/^16^O) relative to the VPDB standard using a within-run laboratory standard calibrated against NBS standards.

### Core-age estimates

Five bulk sediment samples underwent radiocarbon dating at the Natural Environment Research Council (NERC) radiocarbon facility. Radiocarbon analysis on one of these samples, taken at the sediment–water interface from the Glew core, was undertaken to assess any hard water, reservoir, effect in Esmeralda as it is assumed that the age of this layer is the year of coring (c. −66 years BP or 2016 CE). Calibrated dates were calculated (after correcting for the hard water effect) using OxCal v 4.3 employing the standard parameters for continental archives in the northern hemisphere and the calibration curve IntCal20 (Reimer et al. 2020). An age model was created in BACON (Blaauw and Christen 2011, 2013), using an α = 0.6 and leaving the memory strength parameter = 4 (default value).

## Results

### Subfossil gastropod communities

Two (E6 and E9) of the four sample sites in Lake Esmeralda (Fig. 1d) had a large number of subfossil shells (over 100), while sites Ch1 and Ch4 in Chichancanab (Fig. 1c) had over 50 subfossil shells. Only these sites were considered for this study as they were more likely to provide representative samples (Escobar et al. 2010).

**Fig. 2:**
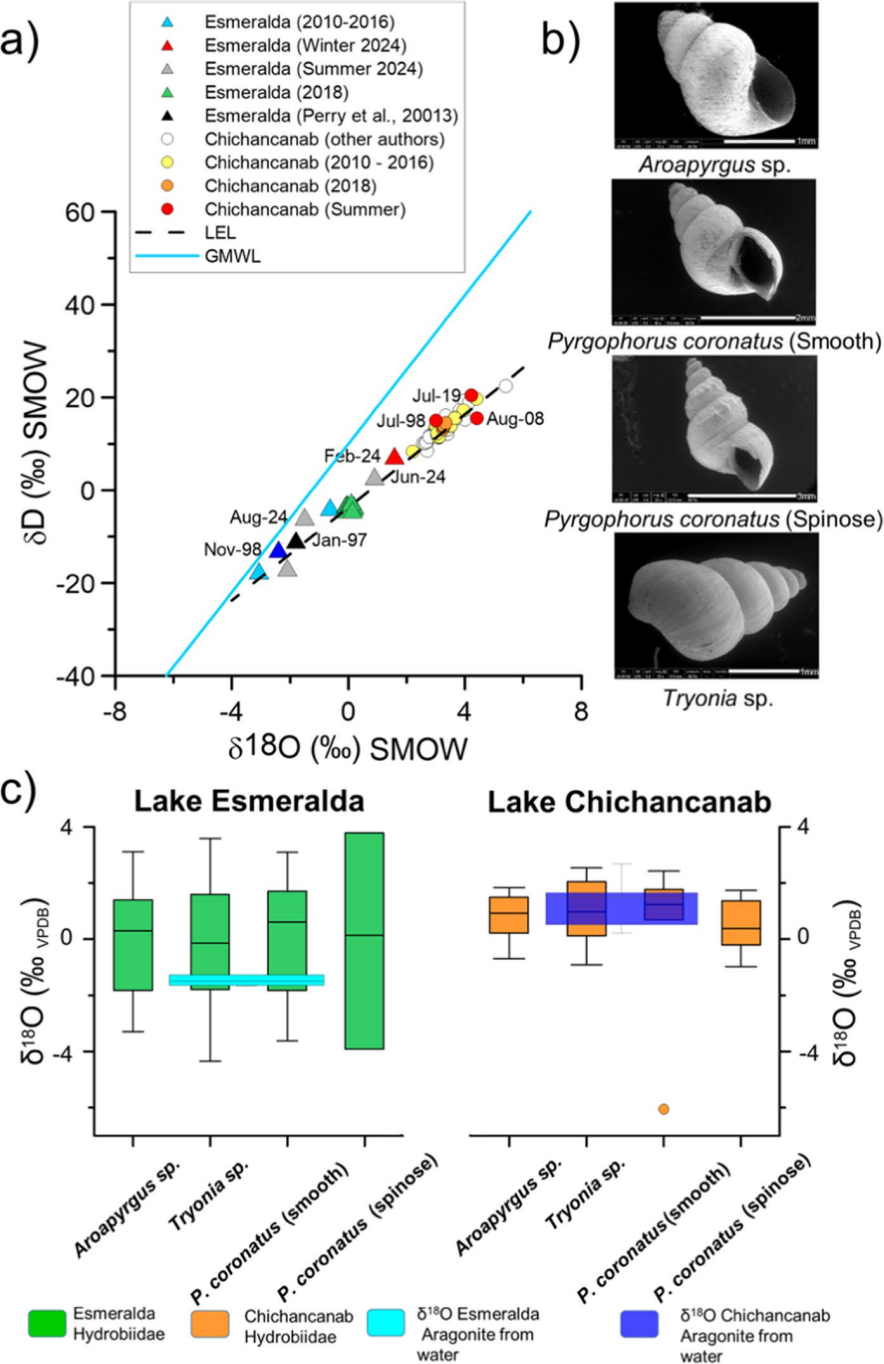
(**a**) Isotopic composition of water samples taken from Lake Esmeralda in January, between 2010 and 2018, compared with samples taken from Lake Chichancanab over the same years. The plot also shows the isotopic composition of water samples collected in the dry season, in winter by other authors in Chichancanab (Covich and Stuiver 1974; Hodell et al. 2012; Evans et al. 2018) and by Perry et al. (2003) in Esmeralda. Summer samples collected in Chichancanab are taken from Cejudo et al. (2022a, b), Douglas et al. (2014) and Perry et al. (2003). The date of collection is indicated for some samples. (**b**) Taxa of the family Hydrobiidae found in lakes Chichancanab and Esmeralda. Images obtained by SEM. (**c**) Comparison between the calculated composition of aragonite from water (blue boxes) and the isotopic composition of shells from different Hydrobiidae taxa in lakes Esmeralda and Chichancanab (green and orange boxes, respectively). Middle line denotes median, box limits denote lower and upper quartiles, box and whiskers denote minimum and maximum values. BGS © UKRI 2026

Three taxa of Hydrobiidae, *Aroapyrgus* sp., *Tryonia* sp. and *P. coronatus*, were found at every site (Fig. 2b). Spinose and smooth forms were observed for *P. coronatus* (Fig. 2b) at sites E6, Ch1 and Ch4.

XRD analyses performed on ten subfossil shells from the different taxa indicated that they are composed of aragonite.

### Isotopic composition of water

The oxygen and hydrogen isotopic signatures of the water samples collected in Lake Esmeralda in 2018 (Fig. 2a, Table 1) have similar values to samples collected in the same season (winter, dry season) from 2010 to 2016 and to samples taken in the dry season during the 1990s (Perry et al. 2003). Some of the 2024 summer samples extend this range along a local evaporation line (Fig. 2a).

**Table 1:** **Temperature** and isotopic composition of surface water and isotopic composition of soluble carbonate (DIC) samples from Lake Esmeralda collected in January 2018

Samples	Water depth (cm)	T (°C)	δ^18^O_Water (‰ VSMOW)_	δD _(‰ VSMOW)_	δ^13^C DIC _(‰__VPDB)_	δ^18^O Calcite _(‰__VPDB)_	δ^18^O Aragonite (‰ _VPDB)_
E1	10	24.2	+ 0.17	−3.68	−8.6	−2.01	−1.41
E2	10	24.1	+ 0.19	−3.19	−9.6	−1.98	−1.38
E3	10	24.4	+ 0.08	−3.66	n. d	−2.15	−1.55
E4	10	24.3	−0.04	−2.94	−6.3	−2.25	−1.65
E5	10	24.2	−0.02	−3.93	−10.0	−2.21	−1.61
E5D	315	n.d	−0.09	−3.16	−8.0	n.d	n.d
**E6**	**10**	**23.6**	**−0.07**	**−3.13**	**−10.0**	**−2.14**	**−1.54**
E7	10	24.3	−0.02	−3.25	−8.2	−2.23	−1.63
E8	10	24.2	−0.06	−4.17	−8.1	−2.25	−1.65
E8D	270	n.d	+ 0.10	−2.55	−11.3	n.d	n.d
**E9**	**10**	**24.0**	+ **0.14**	**−4.20**	**−8.4**	**−2.01**	**−1.41**

Resulting, calculated δ^18^O values of calcite and aragonite that would precipitate from these waters; derived from Leng and Marshall (2004). Values in bold indicate the locations used for taking shells for isotope analyses

The isotopic composition of our water samples collected from Chichancanab (Table 2) from 2009 to 2017 and samples from 2018 also fall on the local evaporation line; however, they have more positive values (c. 4‰ higher in δ^18^O) than Lake Esmeralda (Fig. 2a). Our water isotope results are consistent with the values for Chichancanab (also from dry season, winter samples) reported by Covich and Stuiver, (1974), Hodell et al. (2012), and Evans et al. (2018), and in Esmeralda by Perry et al. (2003). Summer samples collected in Chichancanab by Cejudo et al. (2022b), Douglas et al. (2014) and Perry et al. (2003) have similar isotope values to winter samples (Fig. 2a).

**Table 2:** Temperatures at time of sampling and isotopic composition of surface waters from Lake Chichancanab (all data were collected during December-January, in the dry season)

Field season year	T (°C)	δ^18^O_Water (VSMOW)_	δ^18^O_Calcite (VPDB)_	δ^18^O_Aragonite (VPDB)_
2018	24.3	+ 3.24	+ 1.03	+ 1.63
2018	24.3	+ 3.25	+ 1.04	+ 1.64
2018	24.3	+ 3.28	+ 1.07	+ 1.67
2018	24.3	+ 3.28	+ 1.07	+ 1.67
2018	24.3	+ 3.27	+ 1.06	+ 1.66
2018	24.3	+ 3.34	+ 1.13	+ 1.73
2015–2016	30.5	+ 3.08	−0.35	+ 0.25
2015–2016	29.4	+ 3.11	−0.11	+ 0.49
2015–2016	29.1	+ 3.04	−0.12	+ 0.48
2015–2016	29.0	+ 3.09	−0.06	+ 0.54
2015–2016	29.0	+ 3.12	−0.02	+ 0.58
2015–2016	28.9	+ 3.04	−0.09	+ 0.51
2014–2015	26.2	+ 3.00	+ 0.41	+ 1.01
2012–2013	27.3	+ 3.64	+ 0.83	+ 1.43
2011–2012	n.d	+ 3.52	n.d	n.d
2010− 2011	26.1	+ 3.66	+ 1.09	+ 1.69
2009–2010	24.7	+ 4.40	+ 2.11	+ 2.71
2008–2009	n. d	+ 3.96	n.d	n.d

The theoretical values of calcite and aragonite were calculated based on these data

The δ^13^C values of DIC from Lake Esmeralda (Table 1) are mainly between −11.3 and −8.0‰, with site E4 giving a value of −6.3‰.

### Comparison of water and shell isotope values

Calculated isotope values for theoretical aragonites precipitated from the lake waters (Tables 1 and 2) are similar to the mean measured δ^18^O values of shells from the different Hydrobiidae species obtained in Chichancanab and overlap with the values obtained from Esmeralda (Fig. 2c), although not close to the mean.

The δ^13^C values of DIC from Esmeralda (Table 1) are more negative than the δ^13^C values from the gastropods (Supplementary Figure).

### Downcore isotope values from shells and sediment

**Fig. 3:**
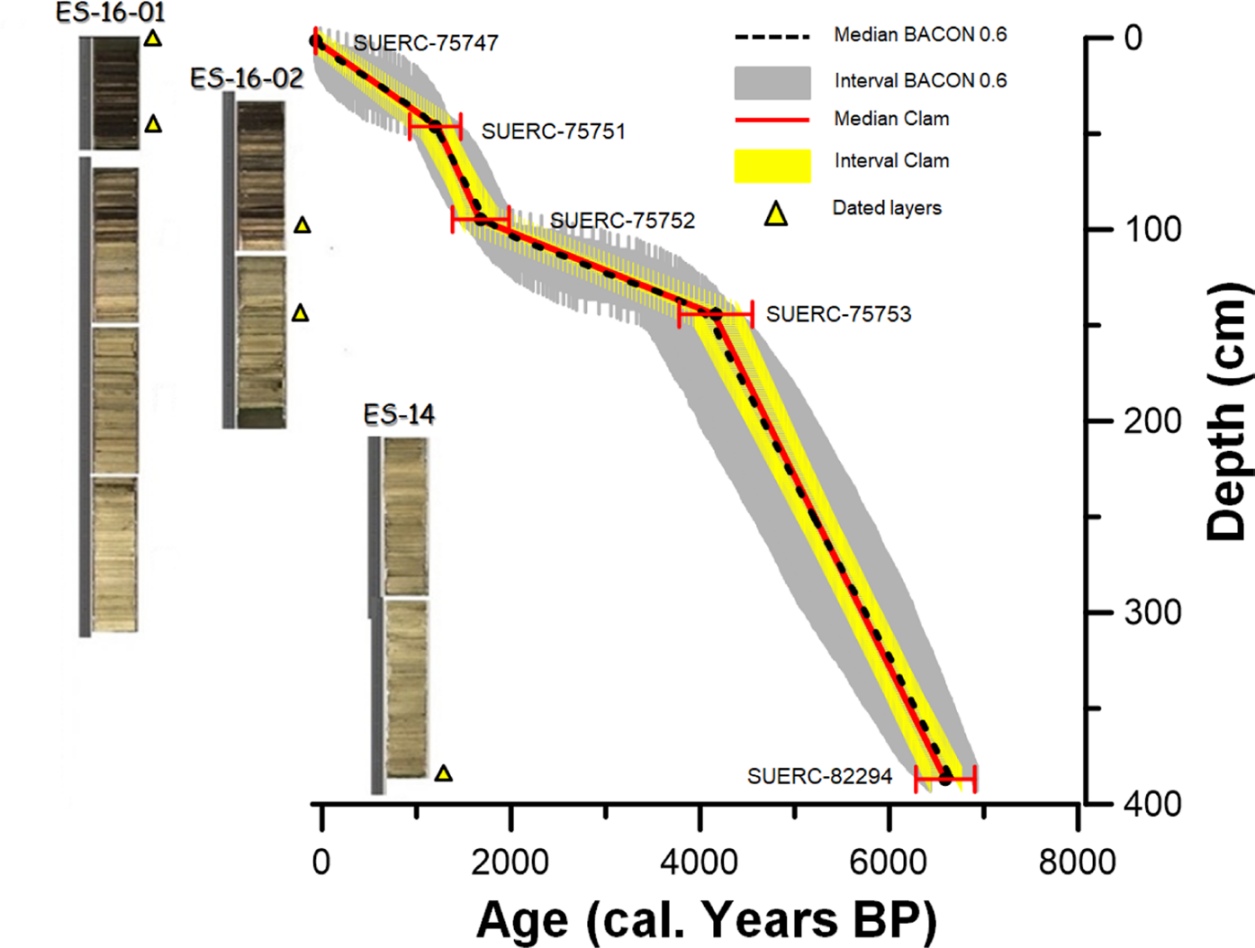
Age model for the sediments from Lake Esmeralda. Left: Stratigraphic correlation between cores ES-14, ES-16-01 and ES-16-02. The yellow triangles indicate the stratigraphic layers where a sample was taken for radiocarbon analysis. Codes for the radiocarbon samples were assigned by the NERC radiocarbon laboratory. Right: Age model used in this research, calculated using an α = 0.6 in BACON 2.2 in comparison to our model on CLAM

The 5 radiocarbon age estimates from the 3.87 m master core (Table 3) show the sequence spans the last c. 6,600 years. The resulting age model (Fig. 3) assumes a constant lake reservoir effect throughout the core of 330 ± 35 years, based on the age of the surface sediments. Sedimentation rates are higher in the lower (below 144 cm), more carbonate rich, section of the sedimentary profile (Fig. 3).

**Table 3:** Radiocarbon dates from Lake Esmeralda. Dating was carried out using the Accelerator Mass Spectrometer (AMS) technique at the Natural Environment Research Council (NERC) radiocarbon facility

Publication Code	Nature of sample	Stratigraphic position (cm)	Layer at Core-Drive	^14^C Enrichment (% Modern ± 1 σ)	Conventional Radiocarbon Age (years BP ± 1 σ)	Corrected lake reservoir effect Conventional Radiocarbon Age (years BP ± 1 σ)	OxCal 2σ youngest age (cal. years BP)	OxCal 2σ oldest age (cal. years BP)	δ^13^C_VPDB_‰ (± 0.1)
SUERC-75747	Bulk sediment	0–3	Water−Core Interface	96.77 ± 0.42	264 ± 35	−66	−66	−66	−27
SUERC-75751	Bulk sediment	47–48	Inner core	81.64 ± 0.37	1630 ± 37	1300 ± 72	1062	1316	−23.5
SUERC-75752	Bulk sediment	93.5–95.5	Inner core	77.16 ± 0.36	2083 ± 37	1753 ± 72	1515	1828	−25.9
SUERC-75753	Bulk sediment	144.5–145	Inner core	60.02 ± 0.28	4101 ± 37	3771 ± 72	3971	4405	−20.8
SUERC-82294	Bulk sediment	385–386	Inner core	46.64 ± 0.21	6127 ± 36	5797 ± 71	6440	6747	−21

Calibrated dates were obtained using OxCal and IntCal20. The age at the top of the core is fixed at −66 calibrated years BP, which was the date of coring

**Fig. 4:**
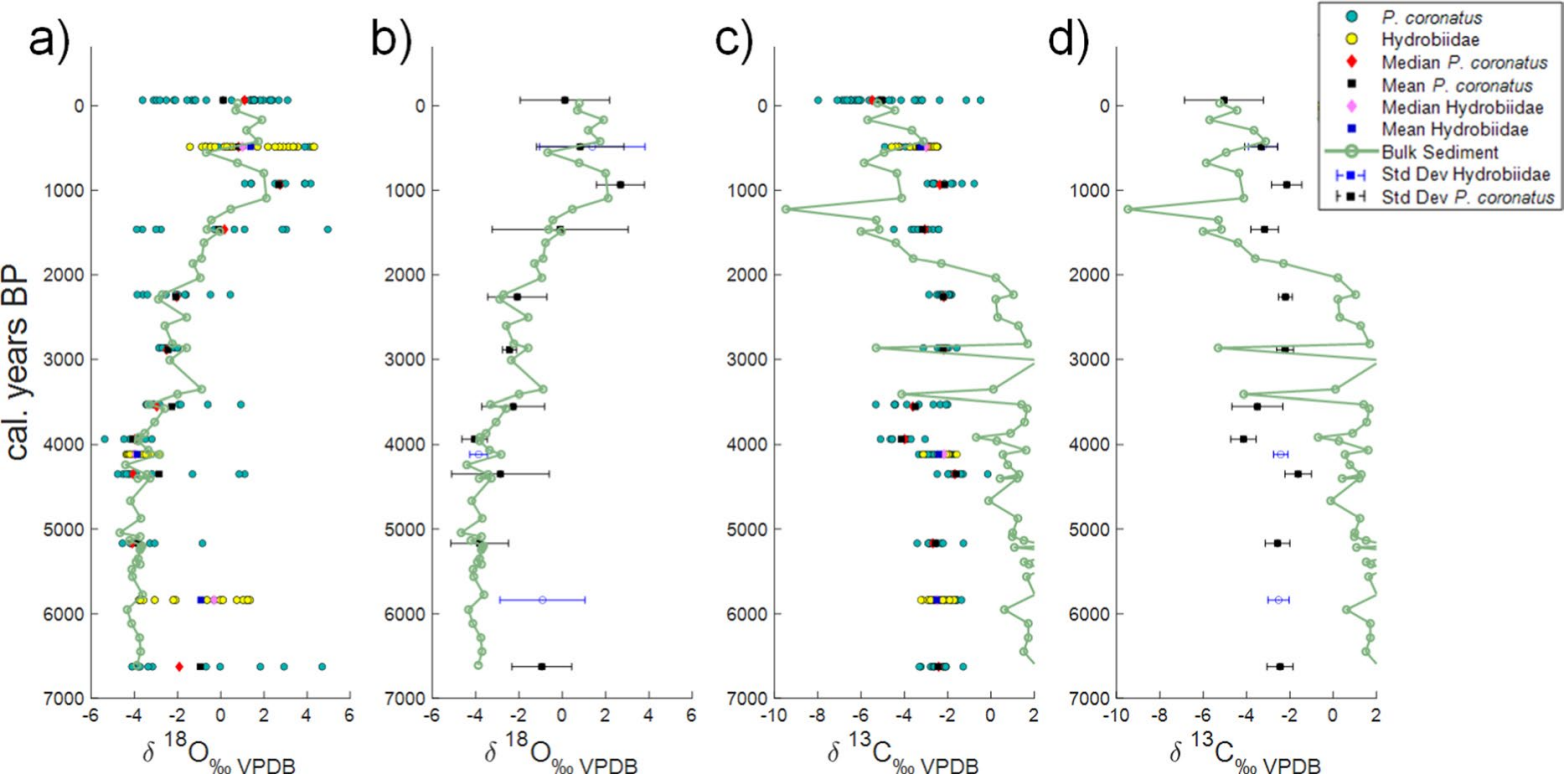
Isotope composition of single shells of P. coronatus from nine levels (or Hydrobiidae in three levels) compared with the low-resolution isotope record developed from sieved bulk carbonate sediments (< 250 μm) sampled every 10 cm in the Esmeralda core sequence. a) The calculated median and means of the δ^18^O values obtained from single shells from each stratigraphic level studied against the δ^18^O record of the fine sediment fraction, b) the mean and the standard deviation of the δ^18^O values of single shells against the δ^18^O values of the fine fraction, c) the median and mean of the δ^13^C values obtained from single shells from each stratigraphic level studied against the δ^13^C record of the fine sediment fraction, d) and the mean and the standard deviation of the δ^13^C values of single shells against the δ^13^C values of the fine fraction. BGS © UKRI 2026

Average δ^18^O values of the bulk gastropod samples from a given level show trends similar to the values from bulk fine fraction sedimentary carbonates through the majority of the core (Fig. 4), other than in the bottom c. 1000 years. Oxygen isotope values in bulk carbonates are steady (around -4‰) from the bottom of the core until c. 4000 cal years BP. The δ^18^O values then get progressively more positive, reaching values of c. + 2‰ just before 1000 cal year BP, and remain at those values until the present day.

Shell δ^13^C values remain similar (c. -2.5‰) through most of the core with a trend to slightly more negative values in the last millennium (Fig. 4). The fine fraction carbonate δ^13^C values show different values and trends. From the base of the core until c. 2000 cal years BP values remain at c. + 2‰, with occasional, substantial, negative excursions. From c. 2000 to 1000 cal year BP there is a trend to more negative values, remaining at values of c. -5‰ until the top of the core (Fig. 4).

## Discussion

### Are gastropod-isotope values in equilibrium with lake conditions?

The water isotope data (Fig. 2) show that Lake Esmeralda is a relatively hydrologically closed system, which is affected by evaporation, although to a lesser extent than Lake Chichancanab. This indicates that Lake Esmeralda probably has a shorter water-residence time than Chichancanab, which could be due to a surface outflow to the north of the basin, which may be seasonal, or because of relatively more groundwater inflows and outflows i.e. compared to lake volume. The dominant evaporative control on these systems means that it is likely that changes in temperature will have less impact on changing carbonate fractionation from lake waters and δ^18^O values in these systems than water balance. This is supported by the range of downcore δ^18^O values (Fig. 4), which is too large (> 10‰) to be realistic due to Holocene temperature changes, which would have needed to be in the order of 40 °C to fully explain the recorded variability (Leng and Marshall 2004).

Gastropod δ^18^O values are in line with theoretical δ^18^O values for aragonite that would have precipitated from lake waters with the temperatures and isotope values measured in this study (Fig. 2), although for Esmeralda at the negative end of the range of measured gastropod values. The greater range of gastropod δ^18^O values in Esmeralda in comparison to Chichancanab may reflect a greater intra annual range of δ^18^O values in the waters of this more hydrologically open system (discussed further below).

The difference between δ^13^C values of modern shells and DIC from the lake water in Esmeralda suggests disequilibrium precipitation, which may be related to fractionation of the DIC isotopes by the gastropods during shell growth, or because there are additional sources of carbon impacting these values, e.g. due to the diet of the snails (Apolinarska et al. 2015b; Covich 2010; Leng et al. 1999).

### Do gastropod-isotope values vary between species?

There are no statically significant differences between the δ^18^O and δ^13^C values of the subfossil Hydrobiidae taxa for either lake (Table 4, see also Supplementary Figure). The shell carbonate precipitates in equilibrium with the δ^18^O values of the host water at these sites, and although there may be fractionation of the δ^13^C signal by the gastropods, there are no significant differences in the fractionation by individual species. This observation suggests that any species from the Hydrobiidae family can be used interchangeably, or combined (Whitmore et al. 1996) for isotope analyses in these systems.

**Table 4:** Summary of statistical tests (ANOVA (A) or Kruskal–Wallis (K) depending on data structure) assessing differences in δ^1^⁸O and δ^13^C isotopic composition across species and sites

Test	Species	Isotope	Comparison	*P*-value
**Within Lake**				
K	All	δ^18^O	Chichancanab all sites	0.340
K	All	δ^13^C	Chichancanab all sites	0.158
K	All	δ^18^O	Esmeralda all sites	0.963
A	All	δ^13^C	Esmeralda all sites	0.510
A	*P. coronatus* (spinose)	δ^18^O	Chichancanab S1 vs S2	0.847
A	*P. coronatus* (spinose)	δ^13^C	Chichancanab S1 vs S2	0.622
K	*P. coronatus* (smooth)	δ^18^O	Chichancanab S1 vs S4	0.967
K	*P. coronatus* (smooth)	δ^13^C	Chichancanab S1 vs S4	0.803
K	*Aroapyrgus* sp.	δ^18^O	Chichancanab S1 vs S4	**0.030**
A	*Aroapyrgus* sp.	δ^13^C	Chichancanab S1 vs S4	**0.036**
A	*Tryonia* sp.	δ^18^O	Chichancanab S1 vs S4	0.624
A	*Tryonia* sp.	δ^13^C	Chichancanab S1 vs S4	**0.018**
A	*Pyrgophorus* sp. (smooth)	δ^18^O	Esmeralda 6 vs 9	0.199
K	*Pyrgophorus* sp. (smooth)	δ^13^C	Esmeralda 6 vs 9	**0.002**
K	*Aroapyrgus* sp.	δ^18^O	Esmeralda 6 vs 9	0.052
K	*Aroapyrgus* sp.	δ^13^C	Esmeralda 6 vs 9	** < 0.001**
A	*Tryonia* sp.	δ^18^O	Esmeralda 6 vs 9	0.784
K	*Tryonia* sp.	δ^13^C	Esmeralda 6 vs 9	** < 0.001**
**Between Lake**				
K	*Pyrgophorus* sp. (smooth)	δ^18^O	Chichancanab vs Esmeralda	0.481
A	*Pyrgophorus* sp. (smooth)	δ^13^C	Chichancanab vs Esmeralda	** < 0.001**
A	P. coronatus (spinose)	δ^18^O	Chichancanab vs Esmeralda	0.758
A	P. coronatus (spinose)	δ^13^C	Chichancanab vs Esmeralda	0.069
K	Aroapyrgus sp.	δ^18^O	Chichancanab vs Esmeralda	0.197
K	Aroapyrgus sp.	δ^13^C	Chichancanab vs Esmeralda	** < 0.001**
A	Tryonia sp.	δ^18^O	Chichancanab vs Esmeralda	0.153
A	Tryonia sp.	δ^13^C	Chichancanab vs Esmeralda	** < 0.001**

Significant results (*p* < 0.05) are highlighted in bold

Previous studies have also found that mean δ^18^O values can be similar across multiple gastropod species e.g. Apolinarska and Pełechaty (2017) in Lake Lednica, Poland. However δ^13^C values are more species-specific in this system highlighting the need for lake specific work on modern and subfossil shell material where possible ahead of interpretation of down-core records.

Beyond the lack of variation between the isotope values of different species, *P. coronatus* appeared in all core layers sampled for gastropods here, illustrating why this species is often chosen for this kind of study. In comparison, the other two taxa are sometimes absent or only occur in very low numbers. For the subfossil shells there are no significant differences in δ^18^O values for the smooth or spinose types of *P. coronatus* between lakes, or between sites in the same lake (Table 4).

### How do individual gastropod-isotope values compare to multi-shell samples? Variance of isotope values of modern shells

**Fig. 5:**
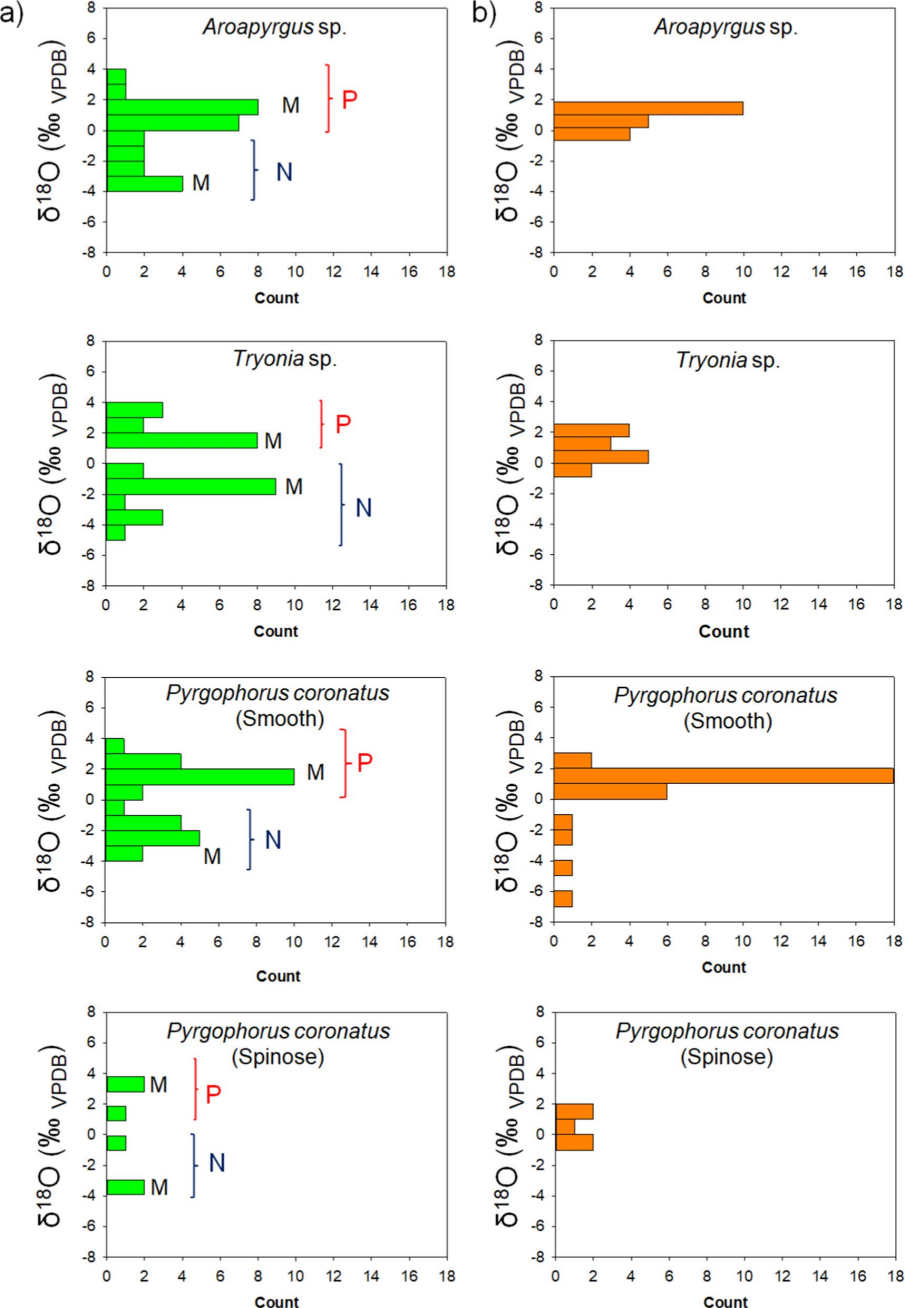
Distribution of the δ^18^O values for the subfossil Hydrobiidae taxa in a) Lake Esmeralda (green) and b) Chichancanab (orange) in bins of 1‰. The groupings of samples M, N, and P are described further in the text. BGS © UKRI 2026

To investigate the variability of subfossil single shell isotope values we plotted the δ^18^O values of each taxon, by lake, in 1‰ bins (Fig. 5). Three of the taxa from Lake Esmeralda show a bimodal distribution (Fig. 5a), and although a similar pattern may exist for *P. coronatus* (spinose), the number of samples is too small to be sure. In contrast, the δ^18^O values of *Aroapyrgus* sp., *P. coronatus* (smooth) and *Tryonia* sp. shells collected in Lake Chichancanab show a skewed unimodal distribution (Fig. 5b).

To investigate the bimodal distribution in Esmeralda further, each group of shells of the same taxon from the same lake was divided artificially into two subsets N (lower δ^18^O values) and P (higher δ^18^O values) according to the closeness of their values to the two local maxima (modes) of the bimodal distribution of the δ^18^O values (indicated by "M" in Fig. 5).

### What could cause the bi-modal distribution?

The lack of a unimodal distribution in the isotope values from modern shells found in the surface sediments in Esmeralda is of particular interest from our analyses here. Building on the discussions of Escobar et al. (2010) on the number of individual shells needed for a significant isotope analysis, the bimodal distribution of modern shells in some, but not all sites e.g. Chichancanab, requires reflection on how this might impact interpretations of past lake conditions.

It is likely that individual shells from the surface samples analysed here, or from an individual core level, will represent multiple years, and/or different parts of a single year. Given this, the bimodal distribution of gastropod δ^18^O values in Lake Esmeralda might be linked to several factors such as (i) the differences between juvenile and adult specimens, or (ii) specimens from different seasons and/or year, and/or (iii) from different parts of the lake.

Differences between juveniles and adult specimens would be reflected in the size and thickness of the shells (Covich 1976, 2010) which are similar for all the samples analysed here. Thus, this potential issue can probably be discounted.

The lifespan of the snails and time of shell growth will impact the δ^18^O values. The study of Drake and Arias (1995) suggested a maximum lifespan of two years for Hydrobiidae, with most living 1.5 years. If this is also true for snails in the Mayab region, then they may preserve a record of seasonal variability in water-isotope values. It is possible that the observed bimodal distribution observed in δ^18^O values in both modern and fossil (core) gastropods in Esmeralda may be explained by seasonal water-isotope variability. Available measurements of modern δ^18^O water values (Fig. 2a) suggest that inter-annual isotope variability in Esmeralda and Chichancanab waters are similar (–2.4 to + 0.2‰ and + 2.5 to + 5.4‰, respectively), but the summer 2024 water samples from Esmeralda suggest an intra-annual range which is as large (> 3‰; Fig. 2). In general, more hydrologically open and smaller systems are likely to see greater variability in their water δ^18^O values than closed/larger systems (Leng and Marshall 2004), although this is not always the case on an intra-annual scale (Jones et al. 2016). The more negative water isotope values, indicative of a more open system, and the greater overall range in gastropod δ^18^O values in Esmeralda (Fig. 2c) both point to a probable greater intra-annual range of lake water δ^18^O values in Esmeralda, compared to Chichancanab, rather than any differences due to the spatial range of lake water δ^18^O values within one lake. This is further supported by the spatial range of only 0.24‰ in the samples collected from different locations in 2018 (Table 1).

The hypothesis of seasonality may get some further support from the similarity between the calculated mean of the δ^18^O values of water from sites in Lake Esmeralda for precipitated aragonite (–1.55‰) (Table 1) and the median values of the artificial subset N of shells of *Aroapyrgus* sp. (−2.14‰), *Tryonia* sp. (– 1.76‰), and *P. coronatus* (–2.08‰) (Supplementary Figure). This resemblance might suggest that subset N was produced in the season when the majority of our water samples were taken. However, subset N is the more isotopically negative of the two subsets, which, would more likely be found in the wetter, summer season, as in 2024. More data on the isotopic composition of water in Lake Esmeralda in summer and transitional months is required to fully test any possible link between the bimodal distribution and seasonal changes in the water-isotope composition of the lake, and how these relate to climate e.g. timing of rainfall and hydrology such as delays in rainfall entering the lake via groundwater.

Water samples from across Esmeralda (Table 1) showed little spatial variability in January 2018, with a range of only 0.3‰. Temperatures were also similar across the lake such that theoretical aragonite values have the same range. This spread of values is an order of magnitude lower than that between the subset N and P, suggesting that spatial variability in lake water isotope values is unlikely to explain the bimodal distribution of the modern shells in Esmeralda. A comparison of the isotope values of shells of the same species collected from different sites (E6 and E9) in Lake Esmeralda shows that the difference in δ^18^O values between the two sites is not statistically significant (Table 4). However, the statistical tests of the δ^13^C values on populations from these two sites showed that the difference in the median values is statistically significant (*P* = 0.003 for *P. coronatus*, *P* =  < 0.001 for *Tryonia* sp. and *Aroapyrgus* sp.). The same difference applies to the samples from Lake Chichancanab (Table 4) where the δ^18^O values are not statistically significantly different between samples across the different taxa, but are for δ^13^C values. This finding again points to a possible dietary, rather than hydrological, control on shell δ^13^C, and is another avenue for future work.

### How do gastropod-isotope values compare with those from fine fraction sedimentary carbonates?

Irrespective of the causes of bimodality in the snail δ^18^O signatures in Esmeralda, the fact that down-core the mean and median values from the gastropods are generally very similar to those from fine bulk carbonates, suggests that the overall drivers of δ^18^O change in Esmerelda are similar for these two forms of carbonate. The similarity of the δ^18^O values from the fine fraction carbonates and mean gastropod values for most of the record suggests that, at least since c. 5500 cal. years BP, Esmeralda sedimentary carbonates are precipitated in the lake, rather than being washed in from the catchment. Both carbonate fractions are recording the same isotope signature (Fig. 2), probably that of changing lake water δ^18^O. The divergence of the two records in the older part of the core sequence suggests that the drivers of δ^18^O change for the two carbonate fractions have not always been the same, especially if the gastropods continued to precipitate carbonate in equilibrium as we have shown them to do at present. In the early part of the core record, Lake Esmeralda bulk carbonates may have derived from the catchment or the controls on the isotopic composition of gastropod shells were different. With a bimodal distribution of gastropod δ^18^O values, the divergence may also be due to changing seasonality over time; where increased seasonality could lead to a greater intra-annual variability in lake water δ^18^O, and in turn allow for a greater difference in sedimentary and gastropod carbonate isotope values precipitated at different times of the year. These differing potential drivers warrant further investigation, particularly as changing climate seasonality in the mid-Holocene has been suggested from other recent studies from the region (Metcalfe et al. 2022).

The relatively small variability in δ^13^C values down core suggests that δ^13^C values in the gastropod carbonate are not coming from the same source as that in the sedimentary carbonate. This could be explained by the gastropods drawing on different sources of carbon (e.g. from dietary or local photosynthetic organisms (Covich 2010; Leng et al. 1999)), rather than the DIC pool more influenced by climate-related water balance change, e.g. residence time (Li and Ku 1997). The difference in δ^13^C values between the snail shells (Supplementary Figure) and DIC in present day Esmeralda (Table 1) support such a hypothesis, but further work is needed to confirm it.

With only 13 layers through the core with direct gastropod to sediment comparison available, this does limit the conclusions we can draw, however the layers analysed with gastropod shells cover the full range of δ^18^O values in the sedimentary carbonates, and are enough to observe a much smaller variability in shell δ^13^C relative to δ^18^O. Our results suggest that fine-fraction lake-sediment carbonate-isotope records from the region can be valuable and that combined records of sediment and shell δ^18^O values can add detail to reconstructions of past lake state as they have been found to do elsewhere (Apolinarska and Hammurlund 2009; Leng et al. 2010).

## Conclusions

This study adds understanding of the isotope signals recorded in gastropod shells, which are a common tool for palaeohydrological reconstructions in lakes, particularly in karstic areas such as the Yucatan Peninsula.

We have confirmed that the use of both single species and composites of Hydrobiidae species can provide robust δ^18^O and δ^13^C estimates, although the constraints on average values may vary between sites. Given the widespread use of *P. coronatus* as a material source for stable isotope records in the Maya lowlands, the differences between spiny and smooth forms revealed by this study may be of interest to taxonomists and warrant further investigation by the palaeolimnological community. The measurement of multiple individual shells adds to understanding of gastropod isotope signatures, e.g. the bimodal distribution of values measured in modern and subfossil shells from Lake Esmeralda. Although further testing would be useful this bimodality seems most likely driven by the intra-annual isotope variability in Lake Esmerelda, which could be typical of such marginally hydrological closed lakes.

The potential value of fine bulk-carbonate-isotope records, even in areas of carbonate geology such as in the case study here, is also noted and may be valuable where the preservation of individual carbonate microfossils (either snails or ostracods) is inconsistent.

We highlight areas where further work might be useful, for example monitoring of intra-annual δ^18^O variability in lakes, which we were unable to fully investigate here. The differences between long term trends in δ^18^O and δ^13^C values highlighted in the Esmeralda record also suggest avenues for additional research, particularly in more open hydrological systems where changes in water balance may not fully dominate the isotope response.

## Supplementary Information

Below is the link to the electronic supplementary material.

Supplementary file1 (DOCX 82 KB)

## Data Availability

Data not included in the manuscript are available from the authors on request.
