# Supplementary material for: Isotopic composition of individual hydrobiidae gastropods from neotropical lakes Esmeralda and Chichancanab in the Maya Cochuah region, Mexico: implications for palaeolimnological research
Source: J Paleolimnol. 2026 Mar 19;74(2):5. doi: 10.1007/s10933-026-00385-3 (PMC12999685; doi:10.1007/s10933-026-00385-3)
Supplement: Supplementary file 1 — Supplementary file1 (DOCX 82 KB) [file 10933_2026_385_MOESM1_ESM.docx]

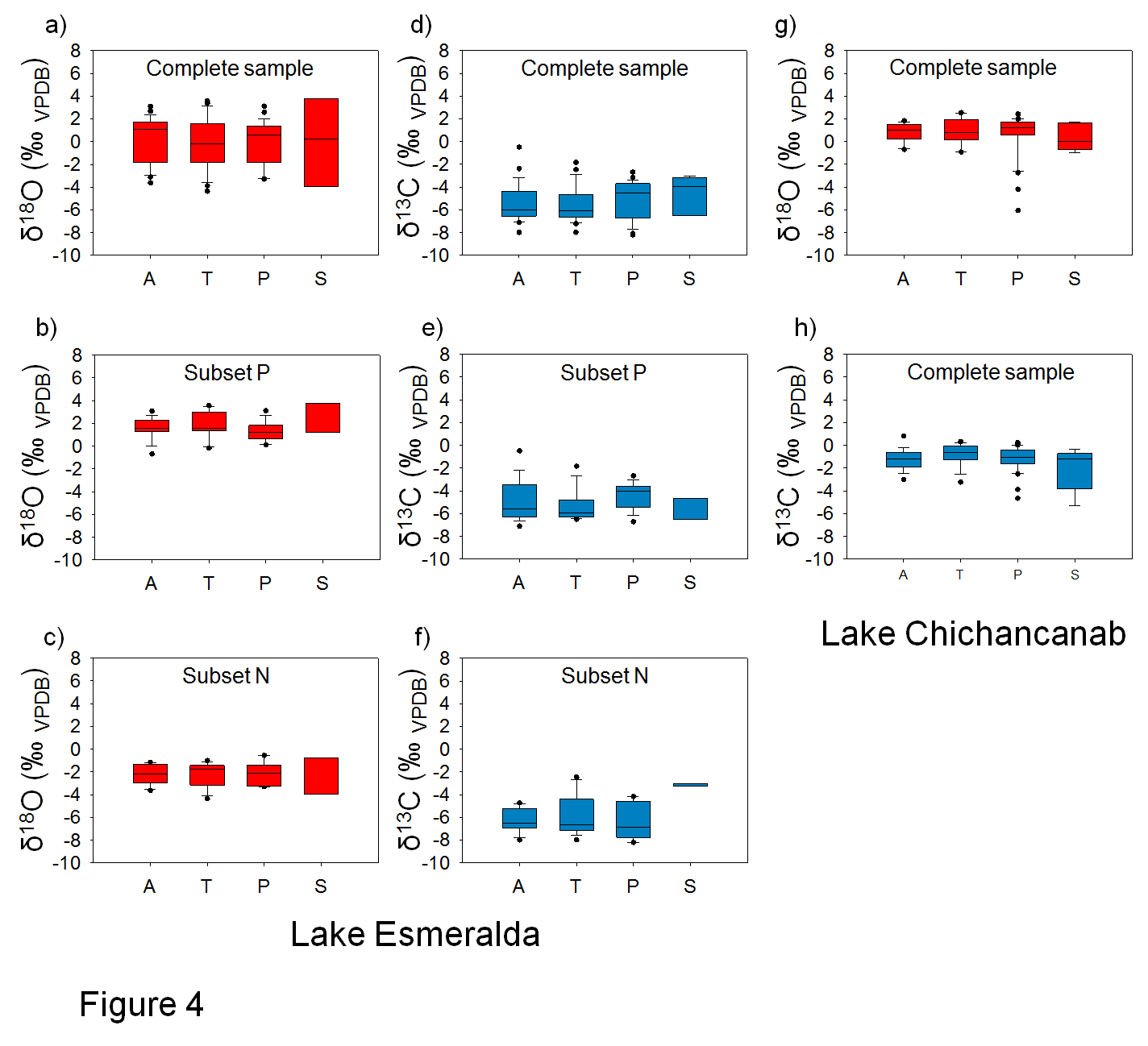


Supplementary Figure Box and whisker plot showing a comparison between the taxa collected at Esmeralda and Chichancanab. a) all δ^18^O (in red) data from Esmeralda. b) δ^18^O values for Esmeralda subset P (see text for details of subsetting); c) δ^18^O at Esmeralda subset N; d) δ^13^C (in blue) at Esmeralda); e) δ^13^C at Esmeralda subset P; f) δ^13^C at Esmeralda Subset N. g) δ^18^O at Chichancanab; h) δ^13^C at Chichancanab. A = *Aroapyrgus* sp.; T = *Tryonia* sp.; P = *P. coronatus* smooth form and; S = *P. coronatus* spinose shape. Middle line denotes median; box limits denote lower and upper quartiles; box and whiskers denote minimum and maximum values. Black points indicate outliers.
